# Bridging the Gap between Statistical and Biological Epistasis in Alzheimer's Disease

**DOI:** 10.1155/2015/870123

**Published:** 2015-05-17

**Authors:** Mark T. W. Ebbert, Perry G. Ridge, John S. K. Kauwe

**Affiliations:** Department of Biology, Brigham Young University, Provo, UT, USA

## Abstract

Alzheimer's disease affects millions of people worldwide and incidence is expected to rise as the population ages, but no effective therapies exist despite decades of research and more than 20 known disease markers. Research has shown that Alzheimer's disease's missing heritability remains extensive with an estimated 25% of phenotypic variance unexplained by known variants. The missing heritability may be explained by missing variants or by epistasis. Researchers often focus on individual loci rather than epistatic interactions, which is likely an oversimplification of the underlying biology since most phenotypes are affected by multiple genes. Focusing research efforts on epistasis will be critical to resolving Alzheimer's disease etiology, and a major key to identifying and properly interpreting key epistatic interactions will be bridging the gap between statistical and biological epistasis. This review covers the current state of epistasis research in Alzheimer's disease and how researchers can bridge the gap between statistical and biological epistasis to help resolve Alzheimer's disease etiology.

## 1. Introduction

Alzheimer's disease is the most common form of dementia and affects as many as 35 million people worldwide [1], and incidence is expected to increase rapidly as the population ages. The reduced cognition and required full-time care that are characteristic of Alzheimer's disease exact a tremendous emotional and financial burden on family members and the health care system. Developing viable therapies is becoming a worldwide necessity.

To date, more than 20 Alzheimer's disease markers have been identified (Table 1), but none have led to the development of effective therapies [1–4]. The majority of these markers were identified using genome-wide association studies, and most are common with small effects on disease risk. In the last several years next-generation sequencing has enabled researchers to sequence whole genomes revealing several rare variants in new genes with large effects [5–8]; however, research suggests that Alzheimer's disease's missing heritability remains extensive [9]. Alzheimer's disease's missing heritability may be explained by missing variants or epistasis, and discovering these genetic factors will require novel study designs [9]. Most studies to date have treated the effect of candidate variants individually. Epistasis in Alzheimer' disease is largely unexplored, but there is compelling evidence that it could play a role in disease [10–13].

Epistasis involves multiple genes contributing to a single phenotype, but the biological nature and implications of epistatic interactions are not always clear. Epistatic interactions are generally discovered in two ways: statistically and biologically. Statistical epistasis is a deviation from additive effects between factors in the model [14], while biological epistasis is a physical interaction between two or more biological components.

Bridging the gap between statistical and biological epistasis is an essential next step towards understanding the genetics of complex phenotypes such as Alzheimer's disease, since most phenotypes in complex organisms involve epistasis. To bridge the gap, we must first understand the benefits and shortcomings for discovering epistasis both statistically and biologically.

Two major challenges for biological epistasis are resources and interpretation. Experiments to discover physical interactions are challenging and expensive to carry out—generally limiting experiments to candidate interactions identified statistically or by some other means. Regarding interpretation, if a physical interaction is discovered only through biological experiments, the implications across phenotypes are often less obvious because discovering an interaction alone does not indicate which phenotypes the interaction affects, thus ultimately leaving questions regarding the biological significance of the interaction. The potential benefits, however, to discovering biological epistatic interactions are substantial. The discovery that two biological molecules interact physically provides crucial functional and pathway information and enables researchers with phenotype information to interrogate a given interaction's role in the phenotype.

Statistical epistasis has its own challenges and benefits. The primary challenge for statistical epistasis is that statistical associations are generally based on genetic variations rather than biological molecules, and the association does not give evidence that the corresponding molecules actually interact physically. In fact, many such statistical associations are based on genetic variations that are not even transcribed (e.g., intergenic) and are not believed to be involved in gene regulation, leaving no obvious biological mechanism for their involvement in known pathways. While this is not always the case, these challenges leave to researchers the arduous task of explaining how the genetic variations, or a nearby gene, could play a role in the phenotype. These explanations often require stepping beyond known biology and sometimes seem implausible. Another major limitation of statistically derived epistasis is the frequency of false-positive and false-negative results. False-positive results are common when testing numerous hypotheses, while false negatives are caused by poor statistical power.

Statistical epistasis can, however, provide insights into unknown biology. For example, just because two proteins are not known to physically interact does not mean they do not both affect the same phenotype; the two proteins may be involved in the same pathway and cause different cascading events, or a given phenotype may be determined by multiple pathways. Such an interaction would be missed in studies of biological epistasis but be discoverable using statistical epistasis. Thus, statistical epistasis can provide the foundation for discovering new biology and generating testable hypotheses. Furthermore, using statistics we can explore whether multiple genetic factors have a nonadditive effect on a phenotype. If so, these genetic factors may be coinvolved in the phenotype's presentation. In this review we discuss the strengths and weaknesses of different approaches for identifying statistical epistasis and review previous studies of epistasis in Alzheimer's disease. Finally, we make suggestions for future studies of epistasis in Alzheimer's disease.

## 2. Methods to Identify Statistical Epistasis: Merits and Limitations

Identifying statistical epistasis is the most common and cost-effective approach to discovering gene-gene interactions, but most studies of genetics in human disease focus on single genetic loci—likely an oversimplification of the underlying biology. To advance our understanding of the genetic architecture of complex phenotypes, we must elucidate the underlying epistatic relationships.

Several analysis methods have been developed specifically to identify gene-gene interactions, including multifactor dimensionality reduction [15–30], log-linear [31] (a form of multifactor dimensionality reduction), logistic regression [10, 11, 32–42], entropy model [43], and the log-likelihood ratio model [44]. Multifactor dimensionality reduction and logistic regression are the two most commonly used methods. Synergy factors are an extension of logistic regression and for the purposes of this discussion are included in that group. Multifactor dimensionality reduction is a nonparametric approach while logistic regression is parametric. Each method has distinct strengths and weaknesses that determine their ability to identify interactions.

Logistic regression has several drawbacks when detecting epistasis: (1) interaction terms grow exponentially as the number of main effects included in the model increases [45]; (2) parameter estimates have large standard errors because the data is high-dimensional—decreasing power to detect interactions [45]; and (3) logistic regression is generally only valid for binary interactions because of limited sample size [12]. Park and Hastie, however, proposed penalized logistic regression as a method to overcome the limitations and showed that penalized logistic regression performs better than multifactor dimensionality reduction in some situations [46].

Many studies have demonstrated the utility of multifactor dimensionality reduction [47–51]. Advantages of multifactor dimensionality reduction include increased power [28, 52] and superior ability to identify high-order interactions even when main effects are statistically insignificant [46]. Multifactor dimensionality reduction, however, is incapable of identifying additive main effects [46] and is less effective if there are missing values in high-dimensional data [45].

Given the complementarity of logistic regression and multifactor dimensionality reduction, combining approaches may be an effective option. For example, multifactor dimensionality reduction could be used to discover complex interactions while logistic regression can be used for main effects.

The prevalence of false positives is a concern for all available methods. According to Page et al. [53], there are four reasons why an allele or interaction between alleles can be associated with a complex disease: (1) it is actually causative; (2) the association is by random chance; (3) a single allele is in disequilibrium with the causative allele; and (4) the association is due to a systematic bias in some portion of the study. Because of the high-dimensionality and small sample size of many studies, there is an increased likelihood of false positives because of the reasons stated by Page et al. “Overfitting” is another potential cause of false positives. Overfitting happens when a complex model is fit to data and is not generalizable beyond the population from which the sample was derived [54]. The cause has commonly been attributed to either genetic or environmental heterogeneity [55], or due to epistasis [14, 56].

There are many approaches designed to prevent false positives and overfitting when studying predictive alleles in a given disease, but they are not foolproof. For instance, protocol when performing multiple comparisons—millions in the case of genome-wide association studies—involves adjusting *P* values to limit the number of false positives due to chance. Similar methods exist to prevent overfitting statistical models to data. Although these methods are useful, researchers mistakenly report false associations.

Even though many weak associations are reported, this practice is not completely wrong. Statistical analyses are limited by the available data, and data is limited by external restraints such as financial support, limited patient availability, genetic material, and even ethical restrictions. Given the various challenges researchers face to produce data, it is not surprising that weak associations are reported. The key to separating true and false associations will be testing in independent data sets if they are large enough, or using meta-analyses across many smaller data sets to determine if the signal is consistent and significant. If a signal is replicable, researchers then need to test associations in cell lines or model organisms.

## 3. Epistasis in Alzheimer's Disease

Numerous studies have identified statistical epistasis in Alzheimer's disease using logistic regression [10, 11, 32–42] and multifactor dimensionality reduction [15–27]. Here we describe studies where results have been replicated in at least two independent samples.

In 2004 Robson et al. identified statistical epistasis between the transferrin* (TF)* C2 allele and the haemochromatosis* (HFE)* C282Y allele using logistic regression and synergy factor analysis [33]. These genes were targeted because of their role in metabolizing iron and previous evidence of iron buildup in Alzheimer's disease patients [57–59]. In 2009, Kauwe et al. replicated the findings from Robson et al. in a separate cohort [34]. There is strong evidence of a biological cascading effect for this statistical interaction, as suggested by Kauwe et al. [34]. HFE binds with transferrin receptor 1 (TfR1), but the C282Y allele has a lesser affinity, allowing TfR1 to bind TF more easily [34, 60]. It was hypothesized that more aggressive binding of TF may cause overabsorption of dietary iron, leading to iron deposits in various tissues [34, 61]. Additionally, Giunta et al. suggested that wild-type TF plays an important role in iron transport and limits amyloid aggregation [34, 62]. All the information supports hypotheses by Robson et al. [33] and Lehmann et al. [63] that this interaction increases Alzheimer's disease risk through increased redox-active iron and oxidative stress.

Likewise, in 2004 Infante et al. identified statistical epistasis between interleukin-6* (IL-6)* and interleukin-10* (IL-10)* associated with decreased risk for Alzheimer's disease based on previous evidence that patients with Alzheimer's disease produce more proinflammatory interleukin-6 and less anti-inflammatory interleukin-10 [64]. In 2009 Combarros et al. replicated the statistical interaction in a separate cohort [10]. This interaction may play a critical role in Alzheimer's disease because Remarque et al. demonstrated that Alzheimer's disease patients have a proinflammatory phenotype and that Alzheimer's disease patients produce more IL-6 (proinflammatory) and less IL-10 (anti-inflammatory) when compared to controls [65]. It is difficult to determine, however, whether this inflammation is contributing to Alzheimer's disease or is simply another side effect of the underlying cause.

In 2009, Combarros et al. performed a comprehensive analysis of over 100 reports of statistical epistasis in Alzheimer's disease, using and introducing their own synergy factor statistic. The synergy factor is a valuable statistic because it relates the expected odds ratio to the observed, summarizing the nonadditive effect of the interaction. This study highlights the innate challenges in discovering statistical epistasis. The authors were only able to support 27 of the originally reported gene-gene interactions using their synergy factor analysis. The challenge with epistatic replication is that there are many factors that influence whether the interaction can be detected in a given data set. Sample size, heterogeneity, and environmental factors are likely the most influential for detecting a real interaction.

In 2014, Gusareva et al. published the first replicable interaction associated with Alzheimer's disease using an exhaustive, genome-wide screening approach [66]. They identified an interaction between* KHDRBS2* (rs6455128) and* CRYL1* (rs7989332) using a cohort from France (2,259 cases and 6,017 controls). The interaction was replicated in a cohort from Germany (555 cases and 824 controls). The interaction was further supported by a meta-analysis using five more independent Alzheimer's disease cohorts. Transcriptome analysis showed decreased expression for both genes in the temporal cortex and cerebellum brain regions. Gusareva et al. hypothesized a biological link between KHDRBS2 and CRYL1 through a potential association with heat-shock proteins and Alzheimer's disease. KHDRBS2 is believed to affect transcription of heat-shock proteins because of studies in its homologue Slm1 in* Saccharomyces cerevisiae* [66, 67]. Slm1 was shown to interact with and activate TORC2 [68], a kinase complex that is part of the TOR pathway, which Pierce et al. demonstrated to affect amyloid *β* and cognitive function in Alzheimer's disease mouse models [69]. Pierce et al. hypothesized that upregulated heat-shock proteins, resulting from inhibition of the TOR pathway, affect amyloid *β* and cognition. This study in particular demonstrates an effective approach to elucidate the functional repercussions of epistasis.

## 4. Epistasis among Top Alzheimer's Disease Genes

Most epistasis studies in Alzheimer's disease involve candidate genes, but to date, few studies [13, 70] have addressed combined effects of the top Alzheimer's disease genes (see Table 1). Verhaaren et al. examined the contribution of the nine AlzGene.org risk alleles to Alzheimer's disease status prediction [70]. They calculated an additive genetic risk score and compared Alzheimer's disease status prediction performance of age, gender, and the apolipoprotein E (*APOE*)*ε4* allele using logistic regression with and without the additive genetic risk score. The genetic risk score did not improve prediction performance significantly, suggesting that the nine alleles may not be diagnostically useful when constrained to an additive relationship. The assumption of additive relationships between risk loci is common but is likely to be an oversimplification of the underlying biology for Alzheimer's disease and other complex diseases [11, 12, 14]. In fact, there may be underlying gene-gene interactions not examined in the Verhaaren et al. study or others that improve Alzheimer's disease status prediction performance.

Ebbert et al. [13] evaluated the possible interactions between the AlzGene.org variants and their effects on Alzheimer's disease in several large, independent data sets. Briefly, Ebbert et al. genotyped each locus in 2,419 subjects from the Cache County Study on Memory Health and Aging to verify results by Verhaaren et al. and explore statistical epistasis among the loci to determine if any interactions are informative to the model in the presence of the main (individual) allele effects. Two interactions were significant in the model: an interaction between* CD33* and* MS4A4E* (*P* < 0.003; SF 5.31, 95% CI 1.79–15.77) and between* CLU* and* MS4A4E* (*P* < 0.016; SF 3.81, 95% CI 1.28–11.32).

## 5. Future Directions

Many researchers are focusing their efforts on epistasis and the community is beginning to discover epistatic interactions that play a role in Alzheimer's disease, but based on odds ratios none of the as-yet discovered interactions appear to play a significant role in Alzheimer's disease etiology. Each of the top candidate genes individually has a consistent and strong signal across numerous data sets, making it a reasonable hypothesis that there are interactions between them. It is not reasonable, however, to assume that the most critical interactions are only between loci with main effects. As such, researchers must approach epistasis in Alzheimer's disease with even larger data sets using exhaustive, genome-wide approaches as demonstrated by the exciting study by Gusareva et al. [66].

The International Genomics of Alzheimer's Project (IGAP) has a data set of over 74,000 cases and controls [4]—a massive data set by today's standards. Given the success by Gusareva et al., a similar agnostic (hypothesis-free) approach in such a large data set would likely result in more stable interactions associated with Alzheimer's disease case-control status, thus leading to potentially useful approaches for both diagnostics and therapeutics. IGAP also discovered several more alleles with main effects in a recent study (Table 1) [4]. Rerunning our analysis across the top loci including IGAP's newly discovered loci may uncover new interactions.

Ultimately, however, we must bridge the gap between statistical and biological epistasis. Biological experiments demonstrating tangible effects on known or novel Alzheimer's disease pathology will be essential to understanding the underlying etiology. These gene-gene interactions may involve physical interactions between proteins, or they may be indirect where they affect a downstream product.

## 6. Conclusions

Epistasis plays a central role in most phenotypes and may play a significant role in Alzheimer's disease. To understand Alzheimer's disease etiology, researchers must utilize both statistical and biological epistasis studies to identify critical interactions and to characterize their functional roles. Some studies have already demonstrated that epistasis plays a role in Alzheimer's disease, but the findings are insufficient to develop effective therapies. By focusing on epistasis and bridging the gap between the statistical and biological knowledge base, researchers will contribute invaluable information for revealing the disease's etiology and developing effective treatments.

## Figures and Tables

**Table 1:** Known Alzheimer's disease associated genes/variants.

Variant	Gene	Abbreviation	Risk/protective
rs744373	Bridging integrator 1	BIN1	Risk
rs11136000	Clusterin	CLU	Protective
rs3764650	ATP-binding cassette, subfamily A (ABC1), member 7	ABCA7	Risk
rs3818361	Complement component (3b/4b) receptor 1	CR1	Risk
rs3851179	Phosphatidylinositol binding clathrin assembly protein	PICALM	Protective
rs610932	Membrane-spanning 4-domain, subfamily A, member 6A	MS4A6A	Protective
rs3865444	CD33 molecule	CD33	Protective
rs670139	Membrane-spanning 4-domain, subfamily A, member 4E	MS4A4E	Risk
rs9349407	CD2-associated protein	CD2AP	Risk
rs9271192^∗^	Major histocompatibility complex, class II, DR beta 5	HLA-DRB5	Risk
	Major histocompatibility complex, class II, DR beta 1	HLA-DRB1	
rs28834970	Protein tyrosine kinase 2 beta	PTK2B	Risk
rs11218343	Sortilin-related receptor, L(DLR class) A repeats containing	SORL1	Protective
rs10498633^∗^	Solute carrier family 24 (sodium/potassium/calcium exchanger), member 4	SLC24A4	Protective
	Ras and Rab interactor 3	RIN3	
rs8093731	Desmoglein 2	DSG2	Protective
rs35349669	Inositol polyphosphate-5-phosphatase, 145 kDa	INPP5D	Risk
rs190982	Myocyte enhancer factor 2C	MEF2C	Protective
rs2718058	NME/NM23 family member 8	NME8	Protective
rs1476679	Zinc finger, CW type with PWWP domain 1	ZCWPW1	Protective
rs10838725	CUGBP, Elav-like family member 1	CELF1	Risk
rs17125944	Fermitin family member 2	FERMT2	Risk
rs7274581	Cas scaffolding protein family member 4	CASS4	Protective

^∗^These SNPs are located close to two different genes so both are listed here (as named in the primary publication reporting the association).
